# Hydroxycinnamic acid amides in rice: biosynthesis, distribution, function, and implication for crop development

**DOI:** 10.3389/fpls.2025.1525268

**Published:** 2025-05-29

**Authors:** Rongrong Xue, Ning Gao, Jing Chen, Zicha Wu, Na Sun, Ying Li, Miuhua Gong, Rensen Zeng, Yuanyuan Song, Dongmei Chen, Jie Wang

**Affiliations:** ^1^ Key Laboratory of Ministry of Education for Genetics, Breeding and Multiple Utilization of Crops, State Key Laboratory of Agricultural and Forestry Biosecurity, College of Agriculture, Fujian Agriculture and Forestry University, Fuzhou, China; ^2^ Key Laboratory of Crop Biotechnology of Fujian Higher Education Institutes, Key Laboratory of Biological Breeding for Fujian and Taiwan Crops, Ministry of Agriculture and Rural Affairs, Fujian Agriculture and Forestry University, Fuzhou, China; ^3^ Key Laboratory of Vector Biology and Pathogen Control of Zhejiang Province, School of Life Sciences, Huzhou Normal University, Huzhou, China

**Keywords:** rice, hydroxycinnamic acid amide, plant defense, metabolite, biosynthesis, environmental stress

## Abstract

Rice (*Oryza sativa* L.) is critical for providing energy and nutrients and ensuring food security for over half of the world’s population. However, like other crop plants, rice is vulnerable to various environmental stresses. To combat these stresses, plants accumulate numerous secondary metabolites known as phytoalexins. Hydroxycinnamic acid amides (HCAAs) are a widely distributed class of phenylpropanoid-derived phytoalexins with diverse biological functions. Increasing evidence highlights their pivotal roles in both abiotic and biotic stress responses, as well as in the modulation of plant growth and development. HCAAs are synthesized by inducible hydroxycinnamoyl transferases acting on the free amines and hydroxycinnamic acids, which provide HCAAs with a variety of metabolic, chemical, and functional capabilities due to diverse combinations among the parent compounds. This review synthesizes current knowledge to emphasize the importance of rice HCAAs, providing a comprehensive examination of their biosynthesis, distribution, biological functions, and regulatory mechanisms, particularly in relation to stress tolerance. Furthermore, the review seeks to further explore beneficial properties of HCAAs, as well as to advance their potential application in genetic breeding to develop elite crops.

## 1 Introduction

Rice (*Oryza sativa* L.) stands as one of the most important crops worldwide, providing nutrients and energy for more than half of the world’s population (Zhou et al., 2002). Plants are sessile and exposed to various environmental stresses, which are undoubtedly critical factors affecting crop production (Chakraborty and Newton, 2011; Deutsch et al., 2018). To cope with environmental stimuli, plants have involved a vast array of defense mechanisms, one of which is the generation of an enormous metabolite arsenal (Wang et al., 2019). Understanding the genetic basis of natural variation of the metabolome in major crops like rice is essential for the quality, reliability, and sustainability of the food supply (Chen et al., 2014). Moreover, the study of abundant metabolites in rice plants contributes considerably to basic rice research and breeding practices (Kang et al., 2019; Mutuku et al., 2019).

The adaptation of plants to harsh environments results in specialized metabolites that assertively enable plants to interact with abiotic and biotic stressors (Zhou et al., 2023). Among these specialized metabolites, the amine-conjugated phenolic compounds, hydroxycinnamic acid amides (HCAAs), have drawn a lot of attention in plant development and defense processes (Bouchereau et al., 1999; Campos et al., 2014; Cho and Lee, 2015; Roumani et al., 2020; Wang et al., 2020a, 2023a). It is formed through the condensation reaction between CoA esters of hydroxycinnamic acids and aliphatic, di/poly-amines, or aromatic monoamines. Accordingly, HCAAs can be broadly categorized into basic and neutral groups. The basic group is hydrophilic and ionizable, comprising aliphatic amines such as putrescine and spermidine. In contrast, the neutral group lacks free amino groups, is water insoluble, and includes aromatic amines such as tyramine, octopamine, and tryptamine (Leonard et al., 2022). It is generally proposed that HCAAs are synthesized in the cytosol and transported into the cell wall, where they undergo peroxidative polymerization mostly upon wounding or biotic attack (Facchini et al., 2002; Hagel and Facchini, 2005; Kang and Back, 2006; Lee et al., 2007). These compounds are widely distributed across plant species and play critical roles in plant physiology, including structural reinforcement, abiotic/biotic stress response, and signaling pathways (Muroi et al., 2009; Macoy et al., 2015; Li et al., 2018; Vogt, 2018; Roumani et al., 2021).

A growing number of studies demonstrate that a wide range of HCAAs accumulated in rice plays a vital role as defensive compounds exhibiting both antimicrobial and anti-herbivore activities (Park et al., 2014; Alamgir et al., 2016; Fang et al., 2022). Beyond their protective functions, HCAAs also contribute to the nutritional value of rice, especially in whole-grain varieties. The antioxidant properties of these compounds enhance the quality of rice by reducing oxidative stress during storage and processing, thereby maintaining the rice’s nutritional integrity. Additionally, rice grains enriched with hydroxycinnamic acid derivatives can offer health benefits when consumed, due to their bioactive properties (Călinoiu and Vodnar, 2018; Leonard et al., 2022; González-Rodríguez and García-Lara, 2024). Both *in-vivo* and *in-vitro* assays have demonstrated the potent antioxidant, anti-diabetic, anti-inflammatory, anti-melanogenic, and cytotoxic properties of HCAAs (Leonard et al., 2022). In traditional medicine, HCAAs, known for their antioxidant properties, have been used to treat skin and digestive disorders and even as anti-cancer agents (Liu et al., 2022; Khawula et al., 2023; González-Rodríguez and García-Lara, 2024). However, HCAAs may play vital roles in rice nutrition, stress tolerance, and growth regulation beyond current understanding, warranting further research.

A recent review has summarized current knowledge regarding HCAAs in maize, including its role in stress responses and its potential for conferring health benefits to human beings (González-Rodríguez and García-Lara, 2024). Both maize and rice are important crops in the grass family, and some key metabolites, including flavonoids and HCAAs, played vital roles in the domestication of maize and rice. However, flavonoids had higher variation in maize than in rice, and more abundant HCAAs existed in rice leaf than maize leaves, indicating that there is interspecific metabolic divergence between these two crops (Deng et al., 2020). Rice has been regarded as a model monocot plant for genomics study due to the smallest genome size among the domesticated cereals, high-efficiency transformation technology, and rich germplasm resources (Jiang et al., 2012; Li Y. et al., 2018). The development of metabolic genome-wide associated studies (mGWAS) provides a useful tool for gene-to-metabolite analysis to understand how rice defends against microorganisms, insects, and other threats through metabolic regulation. This article seeks to underscore the significance of HCAAs in rice development and defense mechanisms. This study aims to provide a comprehensive overview of the major groups of HCAAs, delving into their biosynthesis, chemical structures, distribution, and diverse functions, with a particular emphasis on their contribution to stress tolerance. Additionally, the existing challenges in rice HCAA research and promising opportunities for future investigation would also be discussed.

## 2 Biosynthesis and general characteristic of HCAAs in rice

**Figure 1:**
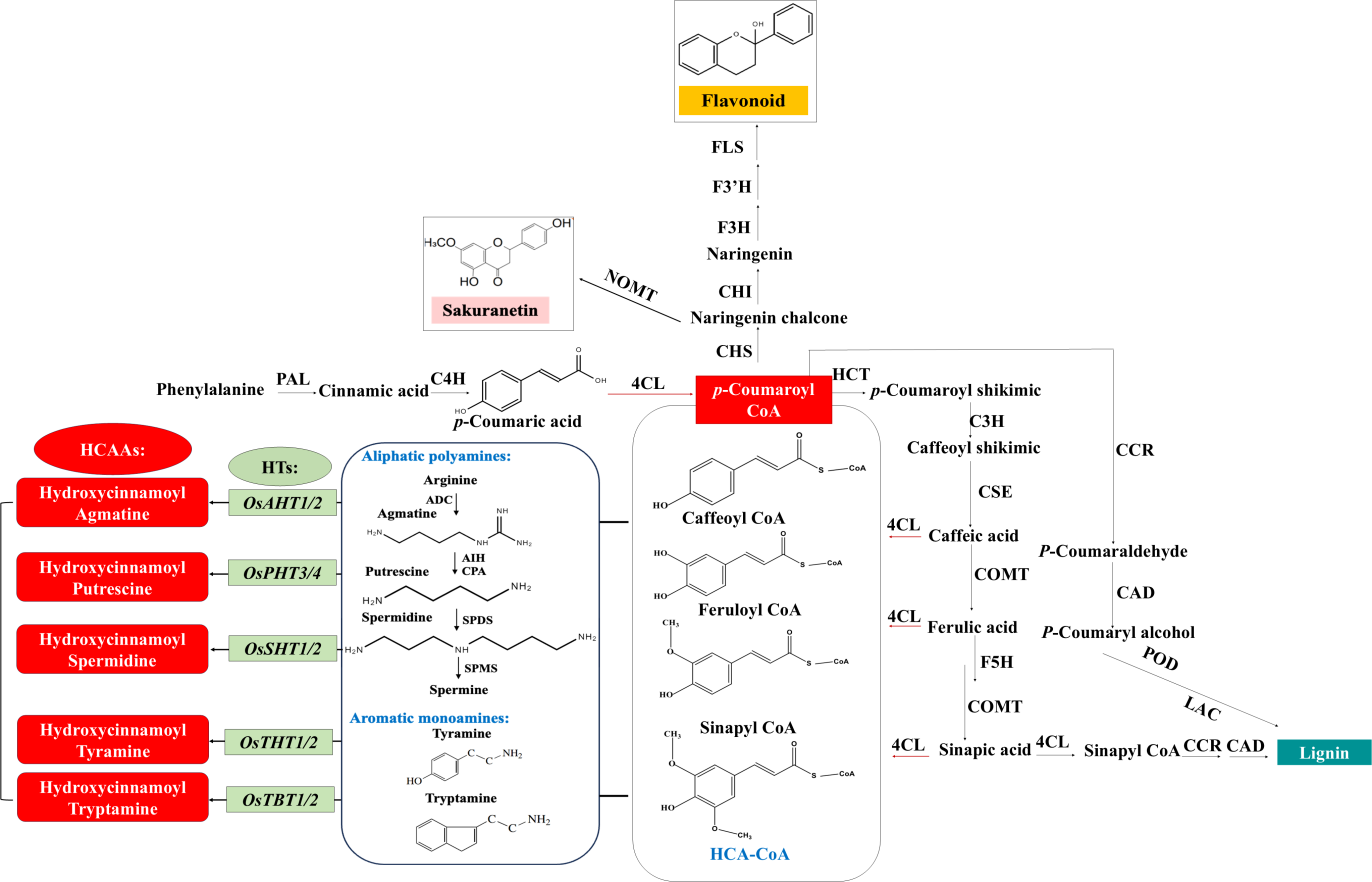
Schematic summary of biosynthesis pathway of the main HCAAs reported in rice. HCAAs are produced by inducible HTs acting on free amine of polyamines or aromatic monoamines and the thioester derivatives of phenolic acids produced through phenylpropanoid pathway, which also regulate the biosynthesis of flavonoid and lignin. Abbreviations of some key enzymes in different metabolic process are indicated. Phenylpropanoid: PAL, phenylalanine ammonia lyase; C4H, cinnamic acid 4hydroxylase; 4CL, 4-coumarate‐CoA ligase; HCT, hydroxycinnamoyl:shikimate/quinate hydroxycinnamoyl transferase; CSE, caffeoyl shikimate esterase; COMT, caffeate/5‐hydroxyferulate 3‐O‐methyltransferase; F5H, ferulate 5‐hydroxylase. Flavonoid: CHS, chalcone synthase; CHI, chalcone isomerase; F3H, flavanone 3‐hydroxylase; F3’H, flavanone 3′‐hydroxylase; FLS, flavonol synthase; NOMT, naringenin 7-O-methyltransferase (Sakuranetin). Lignin: CCR, cinnamoyl‐CoA reductase; CAD, cinnamyl alcohol dehydrogenase; POD, peroxidases; LAC, Laccases. Polyamine: ADC, arginine decarboxylase; AIH, agmatine iminohydrolase; CPA, carbamoylputrescine amidohydrolase; SPDS, spermidine synthase; SPMS, spermine synthase. HCAA: HTs, N-hydroxycinnamoyl transferases; THT, tyramine hydroxycinnamoyl transferase; TBT, tryptamine hydroxycinnamoyl transferase; AHT, agmatine hydroxycinnamoyl transferase; PHT, putrescine hydroxycinnamoyl transferase; SHT, spermidine hydroxycinnamoyl transferase.

HCAAs, also known as phenolamides or phenylamides, are widely distributed types of secondary metabolites in plants and considered as one of the main classes of phenylpropanoid metabolites, along with lignin and flavonoids (Herrmann and Nagel, 1989; Bassard et al., 2010; Dong and Lin, 2021). As illustrated in **Figure 1**, HCAAs are chemical conjugates of various hydroxycinnamic acid esters like coumaroyl-CoA, caffeoyl-CoA, and feruloyl-CoA with aromatic amines like tyramine and tryptamine or polyamines including putrescine, spermidine, and spermine (Edreva et al., 2007; Tanabe et al., 2016). The conjugation connects two different classes of plant protective compounds, spanning the continuum from primary to secondary metabolites, and is catalyzed by those inducible hydroxycinnamoyl transferases (Edreva et al., 2007; Bassard et al., 2010; Macoy et al., 2015). In rice, several genes encoding these transferases have been identified: *Os09g37200* encoding a putrescine hydroxycinnamoyl acyltransferase (*OsPHT*), *Os04g56910* encoding an agmatine hydroxycinnamoyl acyltransferase (*OsAHT2*), and *Os12g27220* encoding a spermidine hydroxycinnamoyl acyltransferase (*OsSHT*) associated with the natural variation in levels of HCAAs were identified based on mGWAS (Chen et al., 2014). Further mGWAS in rice leaf and *in-vivo* metabolic analysis of the transgenic plants identified *Os12g27220* and *Os12g27254* as two *OsSHT* genes that might underlie the natural variation of levels of spermidine-conjugated HCAAs in a collection of rice germplasms (Dong et al., 2015). Further molecular and biochemical studies identified another four genes coding tryptamine/tyramine hydroxycinnamoyl transferases including *OsTHT1/2* and *OsTBT1/2* (Peng et al., 2016). In rice, the levels of aliphatic *p*-coumaroyl putrescine and feruloyl putrescine increase markedly in plants attacked by herbivores and pathogens (Alamgir et al., 2016; Morimoto et al., 2018), and two tandem-duplicated genes encoding putrescine hydroxycinnamoyl acyltransferases were found to be involved in causing the accumulation of HCAAs and the enhancement of HCAAs-dependent resistance against the fungal pathogen *Magnaporthe oryzae* (Fang et al., 2021). Taken together, there are 10 different hydroxycinnamoyl transferases, including *OsAHT1/2*, *OsPHT3/4*, *OsSHT1/2*, *OsTBT1/2*, and *OsTHT1/2*, summarized in **Table 1**, and they can transfer acetyl donors to agmatine, putrescine, spermidine, tryptamine, and tyramine receptors in rice plants, respectively. This process results in the formation of five types of HCAAs in rice, including hydroxycinnamoyl agmatine, hydroxycinnamoyl putrescine, hydroxycinnamoyl spermidine, hydroxycinnamoyl tryptamine, and hydroxycinnamoyl tyramine (Dong et al., 2015; Peng et al., 2016).

**Table 1:** The hydroxycinnamoyl transferase genes identified in rice plants.

Enzyme	Gene	Accession Numbers	Functions	BAHD Family	References
spermidine hydroxycinnamoyl transferases	*SHT1* *SHT2*	Os12g27220 Os12g27254	Induced spermidine-derived HCAAs in rice infested by rice leaf folder, and fall armyworm	Clade V Clade V	(Dong et al., 2015; Zhuang et al., 2022; Xue et al., 2024)
tryptamine hydroxycinnamoyl transferases	*TBT1* *TBT2*	Os11g42290 Os11g42370	Induced tryptamine-derived HCAAs in rice infected by *Magnaporthe oryzae*	Clade IV Clade IV	(Peng et al., 2016; Fang et al., 2022)
agmatine hydroxycinnamoyl transferases	*AHT1* *AHT2*	Os04g56900 Os04g56910	Induced agmatine-derived HCAAs in rice infected by *M. oryzae*	Clade IV Clade IV	(Peng et al., 2016; Fang et al., 2022)
putrescine hydroxycinnamoyl transferases	*PHT1* *PHT2* *PHT3* *PHT4*	Os06g08580 Os06g08610 Os09g37180 Os09g37200	Induced putrescine-derived HCAAs in rice infected by rice brown planthopper, and *M. oryzae*	Clade V Clade V Clade IV Clade IV	(Peng et al., 2016; Fang et al., 2021, 2022; Xu et al., 2021)
tyramine hydroxycinnamoyl transferases	*THT1* *THT2*	Os10g23310 Os10g23820	Induced tyramine-derived HCAAs in rice infected by rice brown planthopper, rice leaf folder, *M. oryzae*, *Xanthomonas oryzae*	Clade IV Clade IV	(Peng et al., 2016; Shen et al., 2021; Xu et al., 2021; Fang et al., 2022)

The majority of hydroxycinnamoyl transferases are members of the BAHD acyltransferase family, and they are able to acylate a variety of metabolites and participate in secondary metabolic reactions (D’Auria, 2006; Dong et al., 2015; Fang et al., 2021). All proteins in this family typically share two conserved motifs, including HXXXD and DFGWG. The HXXXD motif, central to each enzyme, is crucial for catalysis and strictly conserved in BAHD acyltransferases. The DFGWG motif is located near the C-terminal of the protein and might play an important role in the catalytic process and binding of CoA (Xu et al., 2023). The genes of this family are mostly classified into five categories, among which the *N*-hydroxycinnamoyl transferase utilizing branched-chain amines as receptors belongs to Clade V (D’Auria, 2006). In rice, *OsSHT1/2* and *OsPHT1/2* have been identified as members of Clade V (**Table 1**), whereas *OsAHT1/2*, *OsPHT3/4*, *OsTBT1/2*, and *OsTHT1/2* belong to Clade IV (Peng et al., 2016). OsAHT1/2, OsPHT3/4, OsSHT1/2, OsTBT1/2, and OsTHT1/2 can utilize various CoA thioesters, such as feruloyl-CoA, caffeoyl-CoA, sinapoyl-CoA, and coumaroyl-CoA, as acyl donors to catalyze mono-, di-, and tri-acylation reactions with receptor aromatic amines or polyamines, thereby generating a structurally diverse array of HCAA compounds. HCAAs could possess a variety of metabolic, chemical, and functional capabilities due to the large number of possible combinations among the parent compounds (Edreva et al., 2007; Zeiss et al., 2021). Acylation significantly enhances the structural diversity, stability, and bioavailability of HCAAs, thereby contributing to their multiple biological functions in plant growth and development.

## 3 Distribution and breeding implication of HCAAs in rice

Constitutive HCAAs show natural variation and accumulation patterns in rice plant tissues/organs across developmental stages, supporting rice development and stress defense (Dong et al., 2015; Li Z. et al., 2018).

### 3.1 HCAAs distributed in different organs of rice plants

A large amount of basic polyamide-derived HCAAs, especially *N*-trans-feruloyl-putrescine, *N*-trans-(*p*-coumaroyl)-putrescine, *N*-trans-feruloyl-spermidine, and *N*-trans-feruloyl-cadaverine, were accumulated in rice roots (**Table 2**) (Li Z. et al., 2018). Putrescine conjugates, mainly existing as *N*-*p*-coumaroyl and N-feruloyl putrescine, were preferentially accumulated in roots with levels of more than 400 mg/g dry weight (DW). *N*’, *N*”, *N*’”-diferuloyl sinapoyl spermidine exhibited root-specific accumulation of more than 60 mg/g DW. Similarly, a root-specific accumulation pattern also applied to agmatine conjugates (*N*-*p*-coumaroyl and *N*-feruloyl agmatine), while they were detected at much lower concentrations. Additionally, high levels of *N*-trans-cinnamoyl tyramine isolated from the root exudates of a Vietnamese rice cultivar, “OM 5930,” were found to inhibit root and hypocotyl growth of barnyard grass (Le Thi et al., 2014). This indicates that HCAAs may act as potential allelochemicals in rice, inhibiting the growth of neighboring plants or soil microorganisms.

**Table 2:** HCAAs distribution in rice plants.

Compounds	Tissue	References
*N*-trans-feruloyl-putrescine *N*-trans-(*p*-coumaroyl)-putrescine *N*-trans-feruloyl-spermidine *N*-trans-feruloyl-cadaverine *N*-*p*-coumaroyl *N*-feruloyl putrescine *N’*, *N*”, *N*’”-diferuloyl sinapoyl spermidine *N*-*p*-coumaroyl agmatine *N*-feruloyl agmatine	Root	(Li Z. et al., 2018; Le Thi et al., 2014)
*N*’, *N*”-*p*-coumaroyl feruloyl spermidine *N*’, *N*”-diferuloyl spermidine *N*’, *N*”-disinapoyl spermidine	Flag leaf	(Dong et al., 2015)
Feruloyl putrescine *N*’, *N*”-diferuloyl spermidine *N*’, *N*”-disinapoyl spermidine	Panicle	(Dong et al., 2015; Peng et al., 2016)
Di-feruloyl putrescine Di-feruloyl spermidine Feruloyl tyramine Di-feruloyl spermidine *N*-cis-feruloyl-putrescine *N*-trans-feruloylputrescine *N*-trans-(*p*-coumaroyl)-putrescine	Seed	(Bonneau et al., 1994; Li Z. et al., 2018)
Sinapoyl putrescine *N*-coumaroyl spermidine *N*-caffeoyl spermidine *N*’, *N*”-bis(*p*-coumaroyl) spermidine Cinnamoyl-tyramine *p*-coumaroyl-tyramine Caffeoyl-tyramine feruloyl-tyramine Benzoyl putrescine Cinnamoyl putrescine Coumaroyl putrescine Caffeoyl putrescine Feruloyl putrescine Sinapoyl putrescine	Leave	(Tanabe et al., 2016; Fang et al., 2021; Shen et al., 2021; Xue et al., 2024)

In rice flag leaf, *N*’, *N*”-*p*-coumaroyl feruloyl spermidine, *N*’, *N*”-diferuloyl spermidine, and *N*’, *N*”-disinapoyl spermidine were specifically accumulated (Dong et al., 2015). Exogenous application of tyramine to roots significantly promoted the dose-dependent synthesis of coumaroyl tyramine and feruloyl tyramine in young rice leaves, which also suggests that the biosynthesis of hydroxycinnamoyl tyramine in rice is dependent on the availability of tyramine substrate (Lee et al., 2007). In addition, the relatively higher abundance of neutral HCAAs such as conjugated serotonin and tryptamine was also detected in rice leaves (Li Z. et al., 2018). Since rice leaves are frequently attacked by various stressors like pathogens and pests, a large amount of HCAAs were induced in the infested plants besides constitutive HCAAs (Alamgir et al., 2016; Tanabe et al., 2016; Morimoto et al., 2018). Based on these interesting results, the detailed accumulation patterns and regulatory mechanisms of these diverse HCAAs in rice plants under specific stress will be discussed in detail in subsequent sections.

HCAAs have been widely reported to be involved in flower fertility and pollen wall formation in plants (Cabanne et al., 1981; Grienenberger et al., 2009; Quilichini et al., 2015; Roumani et al., 2021). About 80 mg/g DW of *N*-feruloyl putrescine was detected in rice panicles, and two spermidine-derived HCAAs, including *N*’, *N*”-diferuloyl spermidine and *N*’, *N*”-disinapoyl spermidine, were also found abundant in panicles (Dong et al., 2015; Peng et al., 2016). In-depth studies are needed to investigate the role of these HCAAs in modulating rice flowering and productive processes.

Compared to HCAAs distributed in other rice tissues, quite limited levels of HCAAs have been identified in rice seeds. In ungerminated Japonica rice (Tapei 309) seeds, the main amine conjugates are diferuloyl putrescine, diferuloyl spermidine, and feruloyl tyramine, with diferuloyl spermidine making up 50%–60% of the amine conjugate pool (Bonneau et al., 1994). Similarly, only three lower contents of basic HCAAs, including *N*-cis-feruloyl-putrescine, *N*-trans-feruloyl putrescine, and *N*-trans-(*p*-coumaroyl)-putrescine, were detected in rice seeds based on an *in-silico* ultra-high-performance liquid chromatography−high resolution mass spectrometry of HCAAs database (Li Z. et al., 2018). However, these HCAAs were suggested to serve as a storage form of amines involved in the germination process of rice seed through enzymatic hydrolysis, and a sharp decline in HCAAs was observed upon seed germination, with a concomitant increase in polyamines (Bonneau et al., 1994). However, the potential mechanisms of HCAAs constituted as biochemical markers of seed viability require further investigation.

In contrast to previous work showing that rice root contained the highest levels of most HCAAs, followed by flag leaf and panicle, a recent study suggests that rice HCAAs accumulate mainly in panicles, followed by plumules, radicles, leaves, sheaths, stems, roots, and finally seeds (Yang et al., 2022). The distinct distribution patterns of HCAAs in different rice tissues could be associated with rice varieties and techniques used for analysis in different studies (**Table 2**); therefore, more efforts are needed to reveal those unidentified HCAAs, along with their distribution patterns and functions.

### 3.2 The contents of HCAAs associated with rice genotypes

Rice landraces have evolved from their wild progenitor and show high genetic diversity (Huang et al., 2010). The extreme quantitative and qualitative variations in metabolites have been dissected among these diverse varieties, which will provide important insights for breeding elite varieties with increased resistance to detrimental stresses (Keurentjes, 2009; Fernie and Tohge, 2017). HCAAs exhibit variations among different genotypes based on the metabolic data from a diverse worldwide collection of rice varieties (Chen et al., 2014; Deng et al., 2020). For example, two rice varieties, *indica* rice Zhenshan 97 and *japonica* rice Zhonghua 11, were used to quantify the range of variations in HCAAs, and the evaluation revealed significant variation in 10 HCAAs. Among these HCAAs, coumaroylated and/or feruloylated spermidines showed highly subspecies-specific accumulations in *japonica*, while the overall content of coumaroyl or feruloyl agmatine and putrescine was much higher in *indica* than in *japonica* (Dong et al., 2015). In addition, it was found that highly conserved HCAAs showed a higher proportion of differential metabolites in rice leaves compared to those in the seeds (Deng et al., 2020). These studies suggest differences in HCAA abundances among cultivars, which could be further affected by other environmental factors, driving metabolite profile variability in rice. Despite rice as the model plant has been well-studied in genomics, further study is warranted to analyze metabolic disparities among different rice varieties and to reveal the chemical diversity and biological functions of HCAAs in rice adaptation to various environmental conditions.

### 3.3 The contents of HCAAs associated with rice developmental stages

During germination of rice seeds, the amount of HCAA conjugates decreased significantly, while there was a rapid increase in free amine content, which suggests that HCAAs may function as storage forms of amines during germination. Upon hydrolysis, these compounds could provide the cell with additional polyamines, which in turn could affect cell expansion and the viability of the seeds (Bonneau et al., 1994). High levels of sinapoyl putrescine were detected in mature leaves, while feruloyl putrescine was virtually absent in the mature rice leaves (Tanabe et al., 2016). Based on this finding, it is likely that all feruloyl putrescine was converted to sinapoyl putrescine, which appears as the final HCAAs in these mature tissues. Most HCAAs displayed an increase in their levels or accumulated at higher contents at the early stage of development, followed by a rapid decrease in various tissues, except in the root, in which relatively stable accumulation was observed (Dong et al., 2015). Therefore, the degradation of HCAAs could be the way to regulate the pools of bioactive polyamines, which play vital roles in regulating plant growth and defense (Alcázar et al., 2010; Blázquez, 2024). A future detailed investigation should be performed to elucidate the implication of HCAAs in regulating plant development processes.

## 4 Extraction and detection methods for HCAAs in rice

Extraction is a key step in the determination of chemical compounds in rice. Generally, a sample pre-treatment step is required before the extraction. For example, a drying process of rice samples is necessary to stabilize the samples, preventing the microbial spoilage and the hydrolytic rancidity (Chan et al., 2013). In addition, sterilization of rice samples can be conducted through washing with a 1% sodium hypochlorite solution (Mohd. Esa et al., 2013). Finally, the sample is generally ground to a fine powder in order to obtain a homogeneous material, which can be stored in a freezer for further analysis (Goufo et al., 2014). In some conditions, prior to extraction, lipids are removed from the sample by adding hexane (Zhou et al., 2014). Both basic and aromatic HCAAs form ester bonds to structural polymers of the cell wall (Edreva et al., 2007). Sometimes enzymatic hydrolysis with cellulase of rice samples is used to break the bonds between the phenolic compounds and the insoluble polymers of the cell walls to increase the total phenolic content (Wanyo et al., 2014; Sumczynski et al., 2017).

For the actual extraction phase, maceration is the most adopted procedure for the isolation of rice HCAA compounds. Polar solvents like methanol/water or ethanol/water mixtures are used. For instance, acetone/water (70/30, v/v) was used to extract phenolic compounds from rice, and a hydrolysis with acid or NaOH is performed to break the bound phenolics in specific cases (Alves et al., 2016). After impurities from the extract were removed using a filtration membrane, HCAAs in the rice samples were analyzed using chromatographic methods (Chen et al., 2013; Dong et al., 2015; Xue et al., 2024). The selected features of chromatographic methods for the analysis of HCAAs are similar to those of phenolic compounds, which have been well summarized (Ciulu et al., 2018). Therefore, the quali-quantitative profile of HCAAs in rice involves the separation, the identification, and the quantification of the extracted analytes, and HPLC is the most adopted analytical technique.

## 5 Stress-induced the accumulation of HCAAs in rice

In rice, HCAAs are more abundant in leaves, which also show more metabolic interactions than seeds (Deng et al., 2020). Notably, HCAAs accumulate significantly in leaves under environmental stress (Gaquerel et al., 2014; Alamgir et al., 2016; Roumani et al., 2021). Although the general function of HCAAs in plant immunity has been well reviewed (Roumani et al., 2021; Zeiss et al., 2021; Liu et al., 2022), this review will mainly focus on the protective roles of HCAAs in the vital model crop rice, especially under various stresses (**Table 3**).

**Table 3:** HCAA accumulation in rice plants under various stress.

Stress		HCAA	Findings	References
Fungal disease	Rice brown spot fungus (*Bipolaris oryzae*)	feruloyl tryptamine *p*-coumaroyl serotonin feruloyl serotonin	Detectable HCAAs accumulated in *B.oryzae* infected rice leaves. Serotonin and its derived HCAAs incorporated into the cell walls as part of the physical defense against pathogens in rice is associated with the upregulation of the tryptophan pathway.	(Ishihara et al., 2008)
	Rice brown spot fungus (*Bipolaris oryzae*)	*p*-coumaroyl serotonin feruloyl serotonin	(S)-a-(fluoromethyl) tryptophan was utilized to examine the inhibition effects of tryptophan decarboxylase on the defense responses of rice leaves against pathogens including the biosynthesis of HCAAs.	(Ishihara et al., 2011)
	Rice flax spot disease (*Cochliobolus miyabeanus)*	feruloyl putrescine benzoyl tryptamine benzoyl serotonin feruloyl agmatine	FerPut accumulated at the highest concentration, followed by BenTry and BenSer in rice leaves with *Cochliobolus miyabeanus* inoculation Try, Ser, and Tyr-derived HCAAs exhibited weak inhibition of conidial germination, with an inhibition rate of between 5 and 25%.	(Morimoto et al., 2018)
	Rice blast disease *Magnaporthe oryzae*	tryptamine -derived HCAAs	A hydroxycinnamoyl tyramine gene cluster was identified to contribute to the enhanced resistance of rice to against *M. oryzae.*	(Shen et al., 2021)
	*Magnaporthe oryzae*	putrescine-derived HCAAs	A monocot-specific hydroxycinnamoyl putrescine gene cluster contributes to rice immunity.	(Fang et al., 2021)
	*Magnaporthe oryzae*	agmatine -derived HCAAs putrescine-derived HCAAs tryptamine-derived HCAAs tyramine-derived HCAAs	As demonstrated for OsPHT4, APIP5 directly binds to the promoters of OsAHT1/2, OsTBT1/2, and OsTHT1/2, repressing their transcription and HCAA accumulation	(Fang et al., 2022)
Bacterial pathogen	*Xanthomonas oryzae*	benzoyl tryptamine cinnamoyl-, *p*-coumaroyl-, feruloyl-, and benzoyl-serotonins cinnamoyl tyramine benzoyl tyramines feruloyl-agmatine feruloyl-putrescine	FerPut accumulated at the highest concentration, followed by BenTry and CinTyr in rice leaves inoculated with *X. oryzae* HCAAs containing Try (except FerTry) exhibited inhibitory activity in a dose-dependent manner on *X. oryzae* growth, and HCAAs containing other amines barely inhibited bacterial growth.	(Morimoto et al., 2018)
	*Xanthomonas oryzae*	tryptamine-derived HCAAs	A hydroxycinnamoyl tyramine gene cluster was identified to contribute to the enhanced resistance of rice plants against *X. oryzae*	(Shen et al., 2021)
Piercing-sucking insects	Rice brown planthopper (BPH) *Nilaparvata lugens*	Coumaroyl-putrescine Feruloyl-putrescine	p-coumaroylputrescine and feruloylputrescine accumulated in response to attack by BPH. Additionally, BPH feeding on 15% sugar solution containing the two HCAAs had a higher mortality compared to those feeding on sugar diet alone.	(Alamgir et al., 2016)
	BPH	Cinnamoyl putrescine Diferuloyl spermidine *p*-coumaroyl agmatine	levels of CinPut, DiferSpe and CouAgm in leaf sheaths of plants infested by BPH gravid females were 4.9, 7.2 and 24.3-fold, respectively, higher than those in leaf sheaths of non-infested plants	(Wang et al., 2020b)
	White-backed planthopper (WBPH) *Sogatella furcifera*	Cinnamoyl putrescine Caffeoyl putrescine Diferuloyl-putrescine dicoumaroyl-spermidine feruloyl-agmatine *p*-coumaroyl-*N′*-feruloyl putrescine sinapoyl putrescine diferuloyl spermidine	WBPH female adult and WBPH nymph infestation had different effects on the biosynthesis of PAs in rice. The levels of 11 of the 12 identified PAs (only DiferPut was absent) in leaf sheaths infested by WBPH female adult were significantly increased. Infestation by WBPH nymph increased levels of only two PAs, FerAgm and CinPut	(Wang et al., 2020b)
	BPH	cinnamoyl putrescine *p*-coumaroyl putrescine bis(*p*-coumaroyl) spermidine	BPH triggering HCAA was attenuated in JA-deficient rice.	(Xu et al., 2021)
	BPH	*N*-feruloyl putrescine	BPH infestation induced levels of feruloylputrescine	(Qiu et al., 2023)
	BPH WBPH	Cinnamoyl putrescine *p*-coumaroylagmatine diferuloyl putrescine diferuloyl spermidine feruloyl agmatine feruloyl tyramine	six phenolamides were significantly higher in BPH or WBPH-infested ko-*rlk* plants than in similarly treated WT plants.	(Han et al., 2023)
	BPH	*p*-coumaroyl putrescine diferuloyl spermidine feruloyl tyramine	The BPH elicited levels of p-coumaroyl putrescine, di-feruloyl spermidine, and feruloyl tyramine, were significantly reduced in *coi2* mutants compared with WT plants	(Wang et al., 2023b)
Chewing insects	Lawn armyworm (*Spodoptera mauritia*) Rice skipper (*Parnara guttata*)	Coumaroyl-putrescine Feruloyl-putrescine	Two HCAAs are elicited by herbivore attack. However, chewing herbivore larvae did not respond to HCAAs in terms of delayed growth or increased mortality.	(Alamgir et al., 2016)
	*Spodoptera mauritia*	*p*-coumaroyl putrescine feruloyl putrescine *p*-coumaroyl-agmatine feruloyl-agmatine sinapoyl-agmatine feruloyl-spermidine	the putrescine/agmatine N-hydroxycinnamoyltransferase transcripts were induced by applying oral secretions were collected from the 4-5^th^ instar *S. mauritia* larvae in the young rice leaves.	(Tanabe et al., 2016)
	Rice leaf folder (LF) *Cnaphalocrocis medinalis*	Cinnamoyl-putrescine Feruloyl-putrescine	The accumulation of HCAAs and trypsin proteinase inhibitor were increased after LF infestation in WT but not in JA mutant plants impaired in JA biosynthesis (*aoc-2*) and signaling (*myc2-5*).	(Zhuang et al., 2022)
	Fall armyworm (FAW) *Spodoptera frugiperda*	*N*-feruloyl putrescine	FAW infestation decreased feruloylputrescine in BPH-infested plants	(Qiu et al., 2023)
	FAW	Spermidine-derived HCAAs	Two spermidine hydroxycinnamoyl transferase genes SHT1 and SHT2 involved in spermidine-derived HCAAs biosynthesis were highly modified by H3K9ac in FAW-infested rice plants. Furthermore, the Ossht1 and Ossht2 mutants exhibited decreased resistance against FAW.	(Xue et al., 2024)
UV radiation		*N*-trans-cinnamoyl tryptamine	UV-induced *N*-trans-cinnamoyltryptamine inhibited the growth of rice brown spot fungus and rice bacterial pathogens	(Park et al., 2014)

### 5.1 Pathogen infection

**Figure 2:**
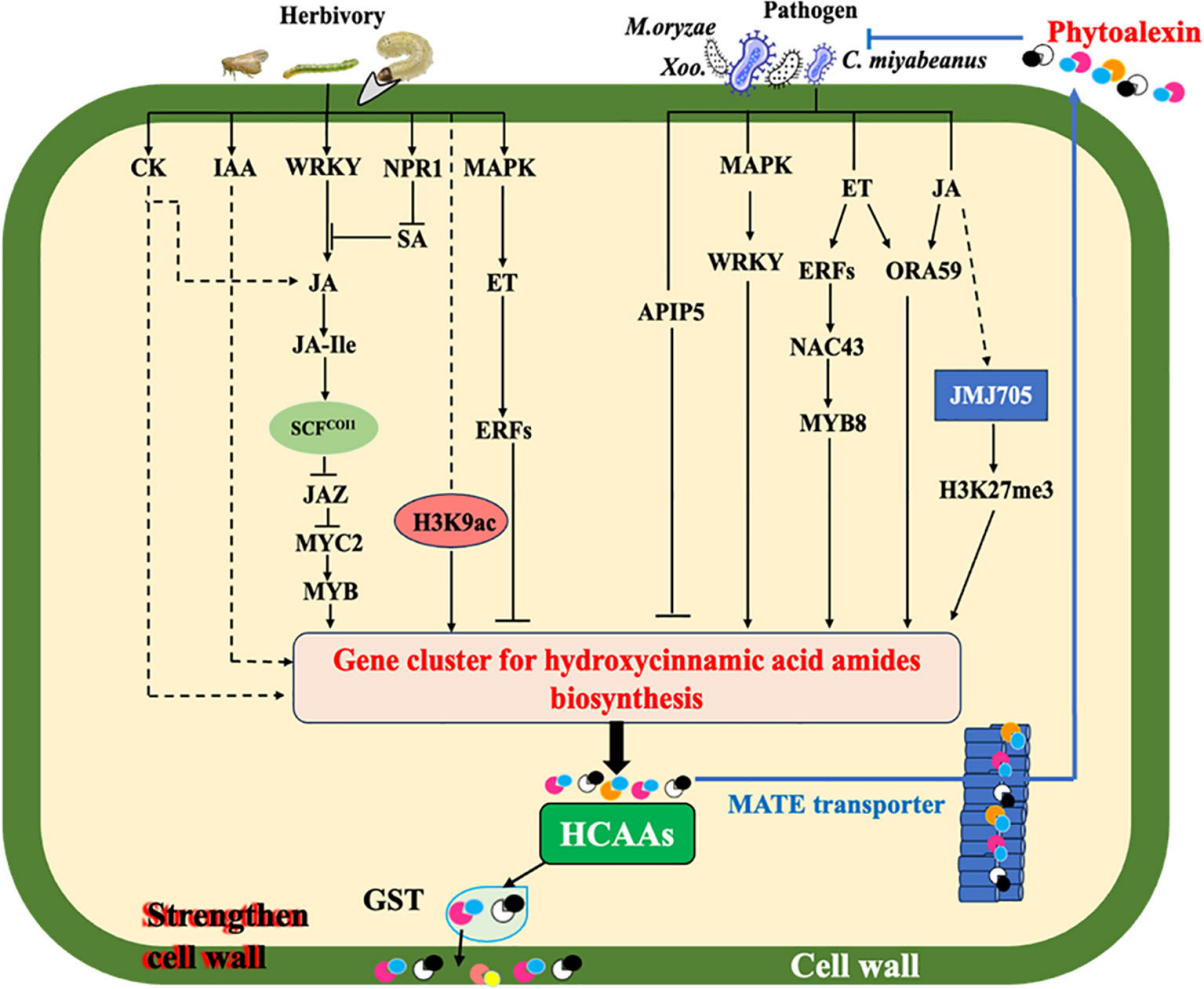
Simplified model of elicitation and signal transduction mediating HCAA biosynthesis under biotic stresses. Solid arrows represent established relationships, while dotted arrows indicate gaps in knowledge. Under pathogen infection, elevation of plant endogenous hormones (JA and ET) stimulates the production of HCAAs. Rapid transcriptional reprogramming by multiple transcription factor genes including WRKYs, ERFs, ORA, NAC, APIP, and MYB of genes encoding HTs for biosynthesis of HCAAs is one of the mechanisms involved in plant defense responses. Glutathione *S*-transferase (GST) may act as an amide carrier protein for HCAAs translocation to the plasma membrane, then deposited on the cell wall. Polyamine and its-derived HCAAs play a vital role in regulating cellular reactive oxygen species (ROS). Under the action of MATE transporter, HCAAs are able to be transported to the leaf surface, thereby inhibiting germination of fungi and bacteria. JMJ705 as a H3k27me3 demethylase could be induced by JA to remove the H3K27me3 from HTs for the biosynthesis of HCAAs during pathogen infection. When plants are attacked by herbivores, the JA signaling pathway is mainly involved through activating coronatine Intensitive1 (COI1) to degrade the jasmonate Zim-Domain (JAZ) proteins and release MYC2, which further activates the expression of MYBs. Herbivory also induces the accumulation of other phytohormones, which can modulate the JA-dependent response and/or contribute to a systemic accumulation of HCAAs. H3K9ac, an epigenetic marker widely distributed in plants, could activate the expression of HTs to synthesis HCAAs in plants under herbivore attacks.

Regarding pathogen infection, as indicated in **Figure 2**, HCAA accumulation mainly relies on phytohormone ET and JA signaling, which activate different transcription factors to regulate the expression of the hydroxycinnamoyl transferase genes (Macoy et al., 2015; Roumani et al., 2021; Liu et al., 2022). Phytoalexins are inducible secondary metabolites possessing antimicrobial activity, and most identified phytoalexins in rice are diterpenoid and phenolic compounds (Cho and Lee, 2015). In addition to the well-known phenolic phytoalexin sakuranetin in rice, some of these induced HCAAs have also been found to exhibit antimicrobial activities, mainly through direct antimicrobial activity or strengthening of secondary cell walls (Roumani et al., 2021; Zeiss et al., 2021). The initial research revealed that serotonin-derived HCAAs, such as feruloyl serotonin and *p*-coumaroyl serotonin, were accumulated in rice leaves infected by fungal pathogens *Bipolaris oryzae* (*B. oryza*) and *Magnaporthe grisea* through regulation of the tryptophan pathway (Ishihara et al., 2008). Additionally, the HCAAs, including cinnamoyl, *p*-coumaroyl, feruloyl, and benzoyl serotonins, benzoyl tryptamine, cinnamoyl, and benzoyl.

Tyramines, feruloyl agmatine, and feruloyl putrescine were induced in rice leaves infected with the fungal pathogen *Cochliobolus miyabeanus* and bacterial pathogen *Xanthomonas oryzae* (Morimoto et al., 2018). Moreover, accumulation of tyramine-derived HCAAs was observed in rice leaves infected with the bacterial pathogen *X. oryzae* and fungal pathogen *Magnaporthe oryzae* (Shen et al., 2021).

A multi-drug and toxin extrusion (MATE) transporter was initially found to export *p*-coumaroyl agmatine on the surface of plant leaves challenged with fungal pathogens (Dobritzsch et al., 2016). Similarly, glutathione *S*-transferase (GST) may act as an amide carrier protein for translocating these HCAAs to the plasma membrane (Macoy et al., 2015). Cinnamoyl tryptamine was found to inhibit mycelial growth of *B. oryzae* with an IC_50_ of 26.92 μg/ml and also exhibited antibacterial activity against *Burkholderia glumae* and *Xanthomonas* sp. with IC_50_ values ranging from 2.45 to 41.09 μg/ml (Park et al., 2014). In addition, both basic and aromatic HCAAs were found to form ester bonds to structural polymers of the cell wall, such as polysaccharides (Edreva et al., 2007; Zeiss et al., 2021). For example, tryptamine-derived HCAAs induced by *B. oryza* infection were reported to be deposited in the cell wall of lesion tissues in rice (Ishihara et al., 2008, 2011). These findings indicate that HCAAs could be transported to directly defend against pathogens or enhance antimicrobial defense through fortifying plant cell walls.

### 5.2 Herbivore infestation

As illustrated in **Figure 2**, insect infestation triggering the accumulation of HCAAs has been reported in a diversity of plants by various herbivore-feeding guilds, including chewing and piercing-sucking insects (Gaquerel et al., 2014; Alamgir et al., 2016; Roumani et al., 2022). Cumulative studies demonstrated that herbivore-induced HCAA accumulation is mainly mediated by the JA signaling pathway (Stitz et al., 2011; Gaquerel et al., 2014). The two HCAAs, *p*-coumaroyl putrescine and feruloyl putrescine, were first reported to accumulate in rice plants subjected to the feeding of chewing insects, such as the lawn armyworm (*Spodoptera mauritia*) and the rice skipper (*Parnara guttata*) larvae, and the sucking insect, the brown plant hopper (*Nilaparvata lugens*, BPH) (Alamgir et al., 2016). Furthermore, the transcripts of putrescine/agmatine *N*-hydroxycinnamoyl transferase genes were strongly induced in the young rice leaves treated with oral secretion (OS) collected from fourth to fifth instar *Spodoptera mauritia* larvae, and these genes were closely associated with the biosynthesis of putrescine/agmatine-derived HCAAs (Tanabe et al., 2016). Furthermore, three HCAAs, including cinnamoyl putrescine, *p*-coumaroyl putrescine, and bis(*p*-coumaroyl) spermidine, were significantly increased in rice plants during BPH infestation (Xu et al., 2021). The levels of all nine examined HCAAs, including putrescine, agmatine, and spermidine-derived HCAAs, were significantly increased in rice plants 48h after rice leaf folder (*Cnaphalocrocis medinalis*) infestation (Zhuang et al., 2022). A recent study in our lab also showed that rice plants infested by fall armyworm (*Spodoptera frugiperda*) larvae had higher levels of spermidine-derived HCAAs compared to those without infestation (Xue et al., 2024).

Four HCAAs, including *N*-feruloyl putrescine, *N*-feruloyl tyramine, feruloyl agmatine, and *N*1, *N*10-diferuloylspermidine, have been reported to reduce the survival rate of female adults of white-backed plant hopper (*Sogatella furcifera*, WBPH) fed on artificial diets supplied with these HCAAs (Wang et al., 2020b). Similarly, BPH insects feeding on a 15% sugar solution containing *p*-coumaroyl putrescine or feruloyl putrescine, at concentrations similar to those elicited by heavy BPH attack on rice plants, had a higher mortality compared to those feeding on a sugar diet alone. However, the growth of chewing insects, *S. mauritia* and *P. guttata* remained largely unaffected by endogenously applied HCAAs (Alamgir et al., 2016). Evidence shows that accumulated HCAAs can significantly inhibit the performance of sucking insects. However, their inhibitory effects on chewing insects have not been consistently observed. Thus, it is crucial to investigate how HCAAs impact the metabolism of various insect species.

### 5.3 Abiotic stress

Studies on HCAAs related to abiotic stress in rice are quite rarer and less detailed than those related to biotic stress, except in studies related to the effects of ultraviolet (UV) on rice plants. UV is an important abiotic stressor that can severely destroy plant DNA and photosynthetic tissues (Demkura et al., 2010). In addition to sakuranetin, which is an important phenolic phytoalexin in rice response to UV irradiation, several HCAAs accumulate in UV-treated rice leaves, including *N*-trans-cinnamoyl tyramine, *N*-benzoyl tryptamine, *N*-trans-cinnamoyl tryptamine, and *N*-*p*-coumaroyl serotonin (Park et al., 2013, 2014). HCAA accumulation has been reported in various plants in response to various abiotic stresses. For example, UV radiation, mineral supplements, high temperatures, and elevation of O_3_ concentration induced the accumulation of putrescine and spermidine derivatives in wheat, as well as in tobacco plants (Königshofer and Lechner, 2002; Edreva et al., 2007; Demkura et al., 2010). In addition, wounding was also found to induce the accumulation of tyramine, octopamine, and putrescine derivatives in tomato (Pearce et al., 1998). In wheat, treatment with CuCl_2_ induced the accumulation of two cinnamic acid amides (Ube et al., 2019). In barley, plants grown at elevated temperature and O_3_ levels accumulated more agmatine-derived HCAAs, which also mediated the resistance to powdery mildew infection (Mikkelsen et al., 2015). As effective free radical scavengers and antioxidants, HCAAs were able to limit the oxidative burst in plants induced by various abiotic stressors (Zeiss et al., 2021; Liu et al., 2022). Further studies are needed to validate the exact roles of these accumulated HCAAs in rice under various abiotic stresses.

## 6 Regulation of HCAA biosynthesis in rice

HCAA metabolism is regulated by multiple mechanisms in response to varying developmental stages and environmental conditions (Roumani et al., 2021). Based on recent advances in rice HCAAs, we summarized potential regulatory mechanisms, including phytohormone signaling regulation, transcription factor regulation, and epigenetic regulation in rice under biotic attacks as examples (**Figure 2**).

### 6.1 Phytohormone signaling

Phytohormones are naturally existing small organic signaling molecules, which play important roles in coordinating responses to various stresses (Altmann et al., 2020). Cumulative data, including studies on mutants and transformations impaired in JA biosynthesis and signaling, demonstrated that HCAA accumulation is mediated by the JA pathway and identified major steps of this pathway (Paschold et al., 2007; Stitz et al., 2011). For example, the accumulation of HCAAs was attenuated in JA biosynthesis (allene oxide cyclase, *AOC*) and signaling (*MYC2*)-impaired JA-deficient lines of rice plants compared with wild-type rice plants during the infestation of BPH and LF (Xu et al., 2021; Zhuang et al., 2022). In addition, rice mutant *Osjar1*, deficient in JA-Ile, was partially affected for herbivore-induced HCAA accumulation, suggesting an alternative regulating pathway such as the ET-signaling pathway (Tanabe et al., 2016). Although the HCAA metabolism is mainly under the regulation of JA signaling, this process can also be modulated by the phytohormone crosstalk with other phytohormones that widely happened in plants (Wei et al., 2021). SA and JA are well-known antagonist phytohormones that promote the defense response to pathogens and herbivores, respectively. In tobacco, NPR1 regulates the antagonistic impact of SA on JA (Rayapuram and Baldwin, 2007). The P2 protein of rice stripe virus obstructs JA-SA crosstalk to facilitate viral infection in rice coordinated by *OsNPR1* (Zhang et al., 2023). In addition, the cytokinin pathway is an important regulator of plant anti-herbivore defense through the accumulation of JA and HCAAs (Schäfer et al., 2015). In *Arabidopsis thaliana*, the expression of agmatine coumaryl transferase genes and HCAA biosynthesis were cooperatively induced by the JA/ET signaling pathways (Li J. et al., 2018). A recent study demonstrated that ET was a local modulator of JA-dependent HCAAs accumulation during *Manduca sexta* herbivory in *Nicotiana attenuate* (Figon et al., 2021). However, some HCAAs, such as cinnamoyl tryptamine, cinnamoyl serotonin, and cinnamoyl tyramine in rice, were not induced by special phytohormones (Morimoto et al., 2018). Further studies are needed to fully understand the mechanism of the phytohormone network functioned in regulating HCAA biosynthesis.

### 6.2 Regulation by transcription factors

Transcriptional regulation plays a central role in the regulation of the biosynthesis of phenylpropanoid metabolites and explains almost all regulatory effects (Yuan and Grotewold, 2020; Huang and Dudareva, 2023). Transcription factors (TFs) are usually regulated at the transcription of multiple biosynthesis genes in a pathway, which makes them attractive tools for improvement of the production of secondary metabolites (De Geyter et al., 2012; Zhou and Memelink, 2016). TFs can integrate internal and external signals to regulate gene expression, thereby controlling the specific accumulation of secondary metabolites (Meraj et al., 2020). Numerous TFs like MYC2, MYBs, bZIP, and WRKYs have been shown to play crucial roles in plant resistance (Buscaill and Rivas, 2014), and we summarized some of these TFs involved in HCAA biosynthesis in rice.

#### 6.2.1 MYC2

Once JA is accumulated, COI1 degrades the JAZ proteins and releases transcription factors belonging to the basic Helix-Loop-Helix (bHLH) family MYC2, which directly regulate the synthesis of various secondary metabolites with anti-herbivore function (Kazan and Manners, 2013; Luo et al., 2023). For example, during a BPH attack, the accumulation of defensive secondary metabolites such as HCAAs and flavonoids was attenuated in *myc2* knockout lines compared with WT plants (Xu et al., 2021). Similarly, the accumulation of HCAAs increased after rice leaf folder (LF, *Cnaphalocrocis medinalis*) infestation in WT but not in JA mutant plants impaired in JA signaling (myc2-5) (Zhuang et al., 2022). As the mutation of the *MYC2* gene would block JA signaling transduction, these results indicate that the biosynthesis of HCAAs in rice plants is close related to the JA-signaling pathway. Recent studies have revealed that MYC2 and JAMYB from a transcriptional cascade that directly regulates phenylpropanoid pathway genes, including *OsPAL6*, *OsPAL7*, and *OsC4H*, leading to the accumulation of HCAAs and enhancing rice resistance to BPH (Liu et al., 2025). In addition, *OsMYC2* has been found to drastically enhance the activity of the promoter of naringenin 7-O-methyltransferase *OsNOMT*, which is a key enzyme for the flavonoid sakuranetin production (Ogawa et al., 2017). In rice plants, both sakuranetin and HCAAs belong to phenolic compounds, which are primarily derived from the phenylpropanoid pathway (Cho and Lee, 2015). However, the detailed role of *OsMYC2* in regulating the biosynthetic enzymes associated with the HCAAs pathway in rice plants has not been fully understood.

#### 6.2.2 MYB

MYB (v-myb avain myeloblastosis viral oncogene homolog) is one of the more abundant classes of transcription factors, which plays important regulatory roles in all stages of rice reproduction and in a wide range of adversity stresses (Jin et al., 2023). The MAMP-responsive MYB transcription factors MYB30, MYB55, and MYB110 were found to activate the HCAAs synthesis pathway and enhance immunity in rice (Kishi-Kaboshi et al., 2018). MYB30 transcripts were also significantly increased in rice plants under BPH attack (Xu et al., 2021). Interestingly, *OsJAZ9* could directly interact with *OsMYB30* and suppress the transcriptional activation of *OsMYB30*, suggesting that *OsMYB30* is also a JA-responsive TF (Lv et al., 2017). In addition, silencing of *StMYB8* impairs the accumulation of HCAAs and flavonoids and reduces the potatoes’ resistance to *P. infestans* (Yogendra et al., 2017). In tobacco, silencing the *NaMYB8* dramatically reduced HCAA levels and plant resistance to the specialist herbivore *Manduca sexta* (Kaur et al., 2010). In addition, MYB8 appears as a master regulator that controls genes encoding three novel hydroxycinnamoyl transferases (*NaAT1*, *NaDH29*, and *NaCV86*) that catalyze the final steps of caffeoyl putrescine and dicaffeoyl spermidine synthesis (Onkokesung et al., 2012). These results indicate the vital role of MYB TFs in regulating the biosynthesis and function of HCAAs, and further studies are needed to investigate the detailed mechanisms of MYBs in regulating HCAAs enrichment in rice.

#### 6.2.3 bZIP

The basic leucine zipper (bZIP) TF APIP5 negatively regulates rice resistance against *M. oryzae* (Wang et al., 2016; Zhang et al., 2022), and nontargeted metabolomics analysis showed that *APIP5*-RNAi transgenic rice plants accumulate a variety of HCAAs, and *APIP5* has been found to directly bind to the promoters of *OsPHT4*, *OsAHT1/2*, *OsTBT1/2*, and *OsTHT1/2*, repressing their transcription and biosynthesis of HCAAs (Fang et al., 2021, 2022). These results suggest that the HT genes are common targets of *APIP5*, while the role of *APIP5* in regulating the transcription of *OsSHTs* has not been evaluated.

#### 6.2.4 WRKY

As similar to MYBs, the TFs of WRKYs are important regulators of biosynthetic metabolites (Schluttenhofer and Yuan, 2015), and WRKYs may be positive or negative mediators of downstream defense mechanisms (Jiang et al., 2017; Javed and Gao, 2023). For example, *OsWRKY45* is a positive regulator of terpene accumulation, which is involved in plant defense against pathogens and herbivores by activating biosynthetic gene expression (Akagi et al., 2014). Similarly, in wheat, silencing the *TaWRKY70* gene not only weakens resistance to *Fusarium graminearum* but also reduces the content of coumaroyl agmatine and coumaroyl putrescine (Kage et al., 2017). In contrast, *OsWRKY62/76* function as negative regulators of biosynthetic defense-related HCAAs and terpenoid metabolites in rice through a metabolomics analysis, and contents of SA and JA were both elevated in knockout lines of *OsWRKY62* and *OsWRKY76* (Liang et al., 2017). The availability of mutants with knockout of *OsWRKYs* is important for further dissection of the regulatory roles of defensive mechanisms in rice plants.

## 7 Conclusion and future perspective

This review provides a thorough analysis of HCAAs in rice plants, with special attention to their varied distribution, biological roles, and biosynthetic mechanisms. HCAAs are crucial for plant defense, acting as a direct toxin to predators and pathogens and contributing to cell-wall cross-linking and reinforcement (Bassard et al., 2010). HCAAs in growing rice panicles and roots likely provide key protection against herbivores and pathogens, as flowering plants are more resistant to herbivory than young vegetative plants (Diezel et al., 2011). In contrast to constitutive HCAAs in flowers and roots, young rice leaves are frequently exposed to various stresses and have shown a strong response through enhanced accumulation of HCAAs (Alamgir et al., 2016; Tanabe et al., 2016). The molecular mechanisms of HCAA biosynthesis were intensively investigated in other plants such as *Nicotiana tabacum* (Figon et al., 2021) and *Arabidopsis thaliana* (Luo et al., 2009). For instance, the induction of HCAAs in response to herbivory was finely controlled by a dominant regulator, MYB8, in *N. tabacum* (Kaur et al., 2010). However, regulatory networks of HCAAs biosynthetic pathways in rice plants under various stresses remain to be further investigated.

Apart from their biological functions in plants, HCAAs exhibit a wide range of health-promoting properties. For example, *N*-(*p*-coumaroyl) serotonin has been found to have antimicrobial properties against pathogenic bacteria, as well as anti-inflammatory and antiatherogenic effects (Takii et al., 2003). Furthermore, their potential to positively impact gut health and lipid metabolism presents significant opportunities for improving human well-being (González-Rodríguez and García-Lara, 2024). Both sakuranetin and HCAAs, renowned as the unique phenolic phytoalexins in rice, have been reported to have antimicrobial properties (Cho and Lee, 2015). A recent study demonstrated that rice under attack by the phloem-feeding brown leafhopper (BPH) produces an antifungal flavonoid, sakuranetin, which targets the yeast-like beneficial endosymbionts of BPH. In addition, disrupting sakuranetin biosynthesis in rice increased the reproductive performance and nutrition of BPH, along with enhancing plant damage (Liu et al., 2023). Thus, we propose that HCAAs could influence the performance of biological organisms through modulating their symbionts and lipid metabolism. Currently, there are limited studies on the bioavailability and biotransformation of HCAAs, which could be hydrolyzed *in vivo* to produce the phenolic acid and amine moieties through the action of enzymes from the host and/or gut microbiota. For instance, agmatine can be freed from these HCAAs *in vivo*, and these compounds may contribute to health benefits partially through agmatine, an important factor in the longevity of its host (Laube and Bernstein, 2017). This indicates that agmatine-conjugated HCAAs may serve as agmatine carriers and display similar bioactivities. HCAAs are now seen more as metabolic intermediates than mere end products (Bassard et al., 2010). Therefore, HCAAs can be hydroxylated and methylated, stored as conjugates, and mobilized when needed.

Plants primed to accumulate high levels of HCAAs can launch a faster, stronger defense response to future infestations, which suggests a connection between preexisting and newly produced defense-related metabolites, indicating a state of readiness (Tugizimana et al., 2019). Plants store defensive secondary metabolites in specialized structures: water-soluble ones in vacuoles and lipophilic ones in latex, resin ducts, trichomes, and glandular hairs (Khare et al., 2020; Al-Khayri et al., 2023). Once plants are attacked by environmental stressors, these metabolites, including HCAAs, could release rapidly from the injured sites and defend against attackers. Epigenetic regulation in plants facilitates defense priming by introducing specific chemical modifications to DNA and histone within chromatin (Lämke and Bäurle, 2017; Jiang et al., 2020). Histone modification is one type of epigenetic regulation of gene expression that occurs during plant development and environmental responses (Dong and Lin, 2021). It has been found that MeJA or powdery mildew treatments upregulated the expression of JMJ705, an H3K27me3 demethylase, which reduced H3K27me3 levels from the four BAHD N-acyltransferases, including TBT1, TBT2, ACT, and PHT3, in the resistant qingke line (Xu et al., 2022). A recent study in our lab showed that the infestation of fall armyworm induced H3K9ac levels from *OsSHT1/2*, which enhanced the biosynthesis of spermidine-derived HCAAs in rice (Xue et al., 2024). Further research is crucial to identify additional histone markers and histone-modifying enzymes and to understand the mechanisms of epigenetic regulation in the HCAA-mediated defense network in plants.

Therefore, using HCAAs to fight herbivores and pathogens is cost-effective for plants and may have influenced plant evolution. Additionally, HCAAs have been widely reported to have health-promoting and pharmacological properties (Roumani et al., 2020; Wang et al., 2020a). Value addition in rice has caused great interest, as it will benefit food security and human health. For example, pigmented rice grains are important food resources for health, as they contain various nutrients and bioactive metabolites, including some HCAAs (Zhao et al., 2024). HCAAs can aid in breeding stress-resistant plants and enhancing human health, with efforts already underway to engineer HCAA production in rice grains (Park et al., 2009). Importantly, current studies on rice hydroxycinnamoyl transferase genes report no phenotypic changes from knocking out or overexpressing these genes (Fang et al., 2021; Shen et al., 2021). These results suggest overexpressing hydroxycinnamoyl transferase genes can boost rice resistance without harming agronomic traits, offering a new genetic resource for better crop resistance. This review aims to provide a foundation for further HCAA research in rice and other cereals and explore their potential in breeding superior varieties for sustainable agriculture.
